# Cross-sectional Study on Medical Attitude Towards Artificial Intelligence Use in Fibromyalgia: Insights From the Annual Thinking Lab on Fibromyalgia Syndrome (ATLAS 2024)

**DOI:** 10.37825/2239-9747.1066

**Published:** 2024-12-06

**Authors:** Marco Cascella, Cosimo Guerra, Rosario De Feo, Valentina Cerrone, Sonia Farah, Piercarlo Sarzi-Puttini, Fausto Salaffi

**Affiliations:** a Anesthesia and Pain Medicine. Department of Medicine, Surgery and Dentistry ‘Scuola Medica Salernitana’, University of Salerno, Baronissi, 84081, Italy; b Department of Medicine, Surgery and Dentistry, ‘Scuola Medica Salernitana’ University of Salerno, 84081, Baronissi, Italy; c Rheumatology Department, Polytechnic University of Marche, Jesi, AN, Italy; d Rheumatology Unit, IRCCS Istituto Galeazzi - Sant’Ambrogio, Milan, Italy

**Keywords:** Fibromyalgia, Artificial intelligence, Survey

## Abstract

**Background:**

The integration of artificial intelligence (AI) in healthcare has the potential to revolutionize clinical practice, particularly in the management of complex conditions such as fibromyalgia (FM). Despite its promise, the adoption of this technology in practice faces several challenges, including limited knowledge and preparedness among healthcare professionals.

**Aim:**

To evaluate the level of knowledge before and after a workshop on AI in FM among clinicians of different disciplines.

**Methods:**

A survey was conducted at the end of the lab. An anonymous 21-item questionnaire was administered to participants.

**Results:**

This survey (n = 26) revealed that while most had extensive clinical experience and some prior exposure to AI, the majority lacked sufficient knowledge and felt unprepared to integrate AI into FM management. Post-congress, perceptions of AI improved for many, but significant barriers remained, including lack of training, resistance to change, and cost concerns. Key benefits identified were symptom monitoring and decision support. Targeted training and technical support were highlighted as essential for effective AI adoption in clinical practice.

**Conclusion:**

Despite a generally positive shift in perception following the congress, many doctors still feel unprepared and lack the necessary knowledge to effectively utilize AI tools. These results underscore the importance of targeted training and support to implement research and facilitate the integration of AI tools in FM and other clinical settings.

## 1. Introduction

Fibromyalgia (FM) is a chronic syndrome characterized by widespread musculoskeletal pain and persistent fatigue, as well as sleep, memory, and mood issues. The clinical spectrum can also include gastrointestinal issues, headaches, and other problems contributing to the complexity of the condition and making it challenging to diagnose and manage effectively [1]. Diagnosing FM often involves a prolonged process due to the absence of specific biomarkers, leading to its identification primarily through the exclusion of other conditions. The complexity of FM’s clinical presentation demands an equally multifaceted approach to treatment, which often includes a combination of pharmacological interventions, physical therapy, lifestyle modifications, and psychological support [2,3].

Artificial Intelligence (AI) refers to the simulation of human intelligence in machines that are programmed to think and learn like humans. AI systems can process vast amounts of data, recognize patterns, and make decisions with minimal human intervention. In healthcare, predictive and generative AI has the potential to revolutionize various aspects of patient care, from diagnosis to personalized treatment plans. AI-based models can be implemented to analyze complex datasets, uncovering insights that might be overlooked through traditional methods. In the context of FM, AI emerges as a promising approach to improve diagnostic accuracy [4,5], treatment effectiveness [6], and overall patient outcomes [7].

Assessing clinicians’ attitudes toward these new technologies is crucial. For example, a survey of clinicians in ophthalmology, dermatology, radiology, and radiation oncology found that most clinicians believed AI would enhance their field. However, concerns about the outsourcing of healthcare to technology companies and the implications of medical liability were significant. Additionally, the survey highlighted education as a priority to prepare clinicians for the integration of AI in healthcare settings [8]. Furthermore, other studies have highlighted several key factors guiding AI adoption in healthcare, including perceived risk, expectations, previous AI experience, and knowledge of AI [9].

In May 2024, the Annual Thinking Lab on Fibromyalgia Syndrome (ATLAS) convened in Gubbio, Italy, bringing together multidisciplinary experts to address the diagnostic, clinical, and management aspects of FM. This event served as a convergence of leading minds in the field, providing a platform for sharing the latest research, clinical experiences, and innovative approaches. During the Lab, an expert led a workshop on AI and machine learning (ML), introducing attendees to cutting-edge AI technologies and demonstrating how these can be integrated into clinical practice to enhance patient care.

### 1.1. Objective

This study was exploratory. We explored insights gathered from ATLAS attendees, offering a comprehensive view of current perceptions, challenges, and advancements in AI’s integration within FM research and management.

## 2. Methods

### 2.1. Ethics

Ethical guidelines, including principles outlined in the Declaration of Helsinki, were strictly followed to guarantee informed consent, voluntary participation, and the secure handling of sensitive data throughout the survey process. Upon completion, responses were anonymized to ensure participant confidentiality. Our investigation followed the Consensus-Based Checklist for Reporting of Survey Studies (CROSS) guideline [10].

### 2.2. Questionnaire

The survey, conducted as a cross-sectional study, was structured to minimize the possibility of error [11] while maximizing results even with a limited sample size [12]. A paper-based questionnaire, consisting of 21 questions in two sections, demographic information and participants’ perception of AI in FM management, was distributed to the participants. Instruments used to measure specific concepts included Likert scales for attitudes towards AI, multiple-choice questions to assess familiarity with AI methodologies, and open-ended questions for qualitative insights. These instruments ensured balanced coverage of participants’ perspectives, combining quantitative and qualitative data on AI’s potential applications in managing fibromyalgia. The questions assessed participants’ baseline understanding of AI before the ATLAS congress and their evolving perceptions afterward.

Topics covered included participants’ familiarity with different AI methodologies, their perceived benefits, and concerns regarding AI integration into clinical practice, and the specific applications they envisioned within their respective fields. To ensure clarity and relevance, the questionnaire underwent rigorous validation and refinement by experts in AI and FM management [11].

The target population included medical professionals from diverse clinical specialties relevant to FM management, with different years of clinical experience and coming from different geographical areas of Italy. No specific eligibility or exclusion criteria were applied, aside from participants’ agreement to complete the questionnaire, as convenience sampling was used to obtain the study sample (Table 1).

### 2.3. Survey administration

The questionnaire was administered in-person during the ATLAS conference. Participants were asked to complete the questionnaire voluntarily after attending specific sessions. Before conducting the survey at the conference, no formal preparatory process such as interviewer training or advertisement was required, as the questionnaire was distributed directly to participants who were already present at the event. The survey was distributed in paper format, and participants had the opportunity to fill it out immediately during the event. The inperson administration allowed for a high response rate, as participants were engaged directly. The timeframe for the survey was limited to the duration of the conference. The data was entered manually into a digital system after the conference. To minimize errors in data entry, a double-checking process was implemented, where two independent researchers reviewed the entered data for consistency. As the survey was conducted in person and on paper, no issues related to multiple participations or online data security were relevant in this context.

### 2.4. Statistics

Descriptive statistics were implemented to summarize participant demographics (age, gender, profession, geographical location, and years of clinical experience). Frequencies and percentages were computed for categorical variables. No missing data was found in the dataset.

No formal sensitivity analysis was conducted for this study due to the nature of the sampling method and the limited sample size.

Subsequently, we performed subgroup analyses to explore differences in AI perception based on participant profession (e.g., rheumatologists vs. physiatrists) and clinical experience (e.g., less than 10 years vs. more than 20 years).

A cluster analysis was conducted to examine patterns in AI knowledge and usage across different geographical regions and professional specializations.

Data analyses were performed using Python (version 3.10.6; Delaware, United States) including the visualization libraries Matplotlib and Seaborns.

## 3. Results

### 3.1. Demographic results

All 26 eligible participants completed the survey, resulting in a 100 % response rate. This high response rate can be attributed to the in-person administration of the survey during the conference, where participants were easily engaged. No duplicate responses or multiple entries were identified.

Most respondents were female (n = 17, 65.4 %). Regarding age distribution, the largest group of respondents was aged 50–59 years (n = 10, 38.5 %). Participants being 60 years and above accounted for 30.8 % (n = 8), while those aged between 40 and 49 years old represented 26.9 % (n = 7). Only one participant reported being between 30 and 39 years old (n = 1, 3.8 %). Specialization among the participants varied. Rheumatologists were the most represented group (n = 10, 38.5 %), followed by physiatrists (n = 8, 30.8 %), anesthetists (n = 5, 19.2 %), and neurologists (n = 3, 11.5 %). In terms of practice settings, the majority of doctors worked in public practice (n = 16, 61.5 %), followed by those in autonomous practice (n = 8, 30.8 %). Only a small number of doctors were in private practice as dependent (n = 2, 7.7 %).

Most clinicians had extensive clinical experience, with 16 doctors (61.5 %) having over 20 years of clinical experience. Nine respondents (34.6 %) had 11–20 years of experience. Geographically, participants from Southern Italy and the Islands were the most numerous (n = 12, 46.2 %), followed by clinicians from Northern Italy, who accounted for 10 participants (38.5 %). Four participants (15.4 %) came from Central Italy.

### 3.2. AI knowledge

Before the congress, nearly half of the participants (46.2 %) had read scientific articles about the use of AI in clinical practice. However, only a small number (15.4 %, n = 4) had implemented any AI tools in their practice. Among these four clinicians, two (7.7 %) used decision support systems, one (3.8 %) used another type of AI tool, and one (3.8%) reported using multiple AI tools (Diagnostic imaging, decision support systems, patient monitoring, chatbots).

**Fig. 1:**
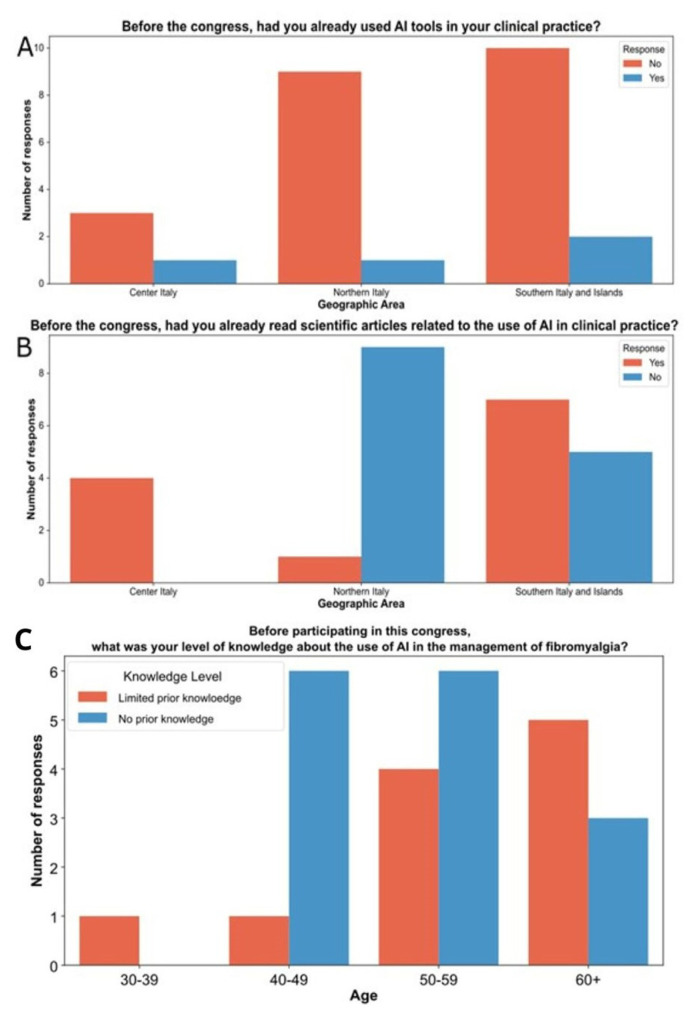
Cluster histogram of artificial intelligence use (A) and knowledge (B) and geographic distribution. Cluster histogram of AI knowledge in fibromyalgia before the congress grouped by age ranges (C).

A majority of participants reported no prior knowledge of AI (n = 15, 57.7 %), while the rest had limited prior knowledge (n = 11, 42.3 %). Regional differences were notable, with doctors from Northern Italy being less likely to have read AI-related articles (n = 9, 90 %) and having the highest proportion with no prior knowledge (n = 8, 80 %) (Fig. 1).

Subsequently, the knowledge of AI implementation in FB was assessed. Only 43 % of clinicians were aware of the potential applications of AI. Age-related differences showed that doctors in the age ranges of 40–49, and 50–59 years, had no prior knowledge in 85.7 % and 60 % of cases, respectively (Fig. 1C).

After the congress, many participants reported an improved perception of AI in FM management (n = 15, 57.7 %), while a smaller group reported a much-improved perception (n = 2, 7.7 %). Some respondents reported no change in their perception (n = 7, 26.9 %), and one doctor reported a worsening view (3.8 %). Notably, 61.5 % of participants felt poorly prepared to integrate AI tools into clinical practice.

In terms of understanding AI, 38.5 % reported gaining a better understanding of the usefulness of machine learning in clinical practice, while 50.5 % reported a partially better understanding. The ability to distinguish between different types of AI algorithms remained low, with 42.3 % unable to distinguish between them and 50 % only partially able to do so. A clearer understanding of neural networks was achieved by half of the participants (50 %), while 42.3 % reported partial understanding. Regarding the use of AI for data analysis and outcome prediction in FB, 53.8 % felt only partially competent, while eight doctors (30.7 %) admitted they were unable to explain it accurately.

**Fig. 2:**
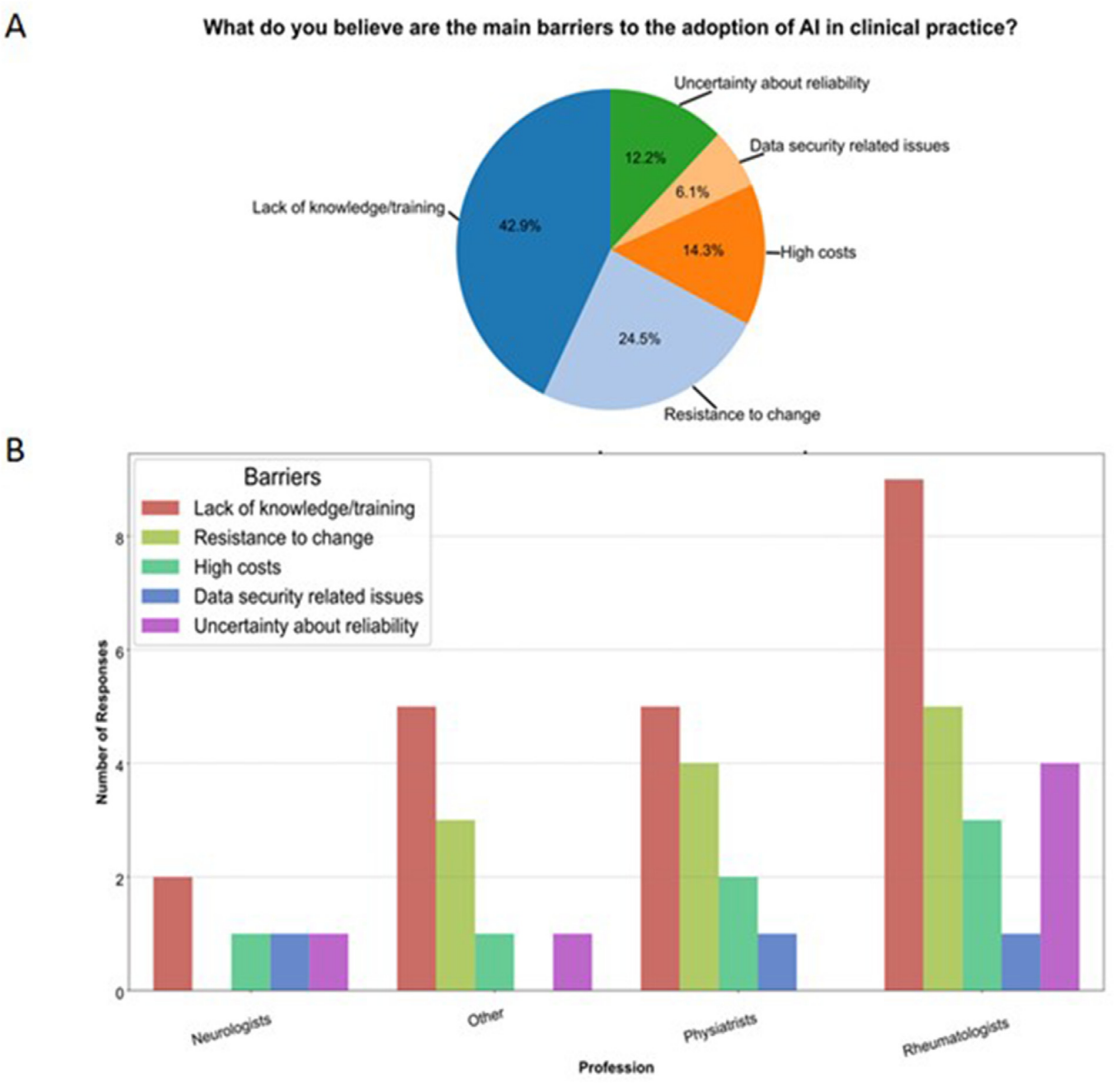
Barriers to the implementation of artificial intelligence in clinical practice (A) and analysis by profession (B).

Perceived barriers to AI adoption varied by profession. Among rheumatologists, the most significant barrier was a lack of knowledge and training (n = 9, 34.6 %), followed by resistance to change (n = 5, 19.2 %), uncertainty about reliability (n = 4, 15.4 %), and high costs (n = 3, 11.4 %). Data security-related issues were not perceived as significant barriers. Other professionals exhibited a similar pattern of responses regarding barriers (Fig. 2).

**Fig. 3:**
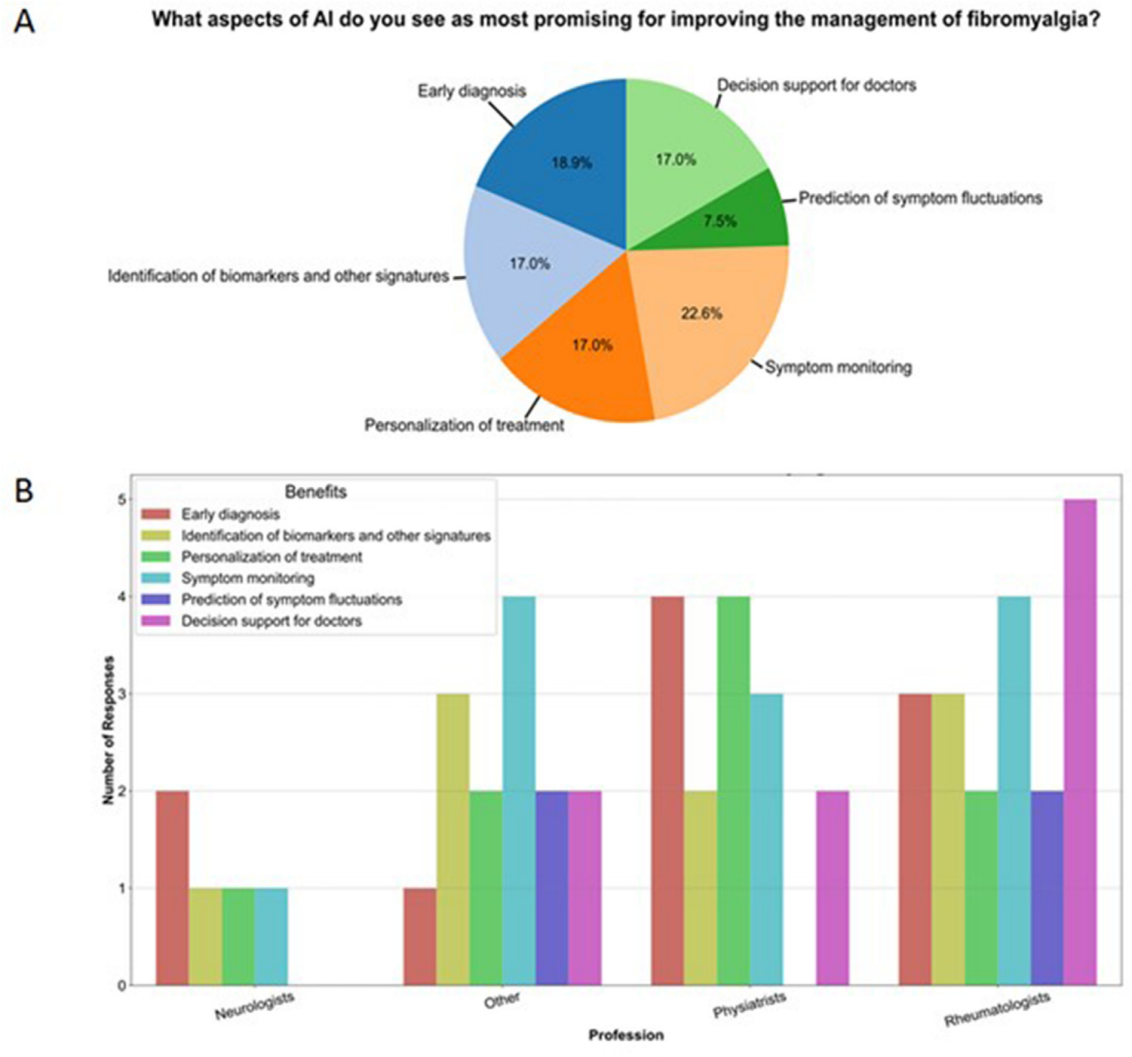
Perceived benefit regarding the use of artificial intelligence in the management of fibromyalgia (A) and analysis by profession (B).

The distribution of perceived benefits of AI was more balanced, with advantages mostly focused on symptom monitoring (22.6 %), and early diagnosis (18.9 %), Rheumatologists particularly highlighted the importance of decision support and symptom monitoring systems (Fig. 3).

**Fig. 4:**
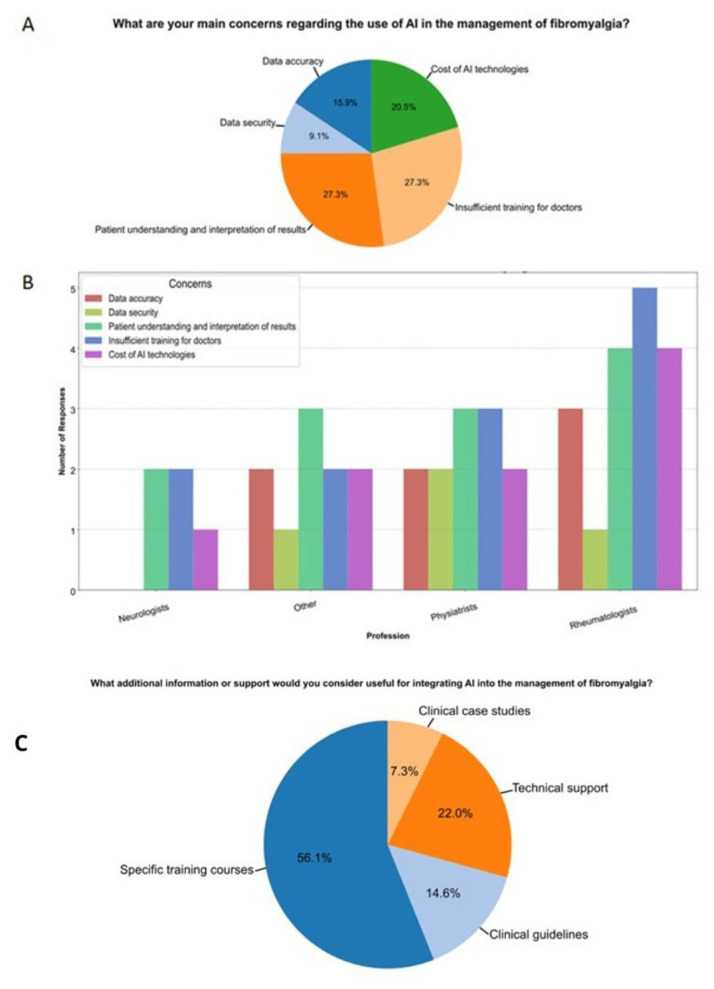
Concerns regarding the use of artificial intelligence (A) and analysis by profession (B). Additional information and supports considered useful by professionals (C).

The most perceived concerns among all professionals were patient understanding and interpretation of results (27.3 %), insufficient training of doctors (27.3 %), and the cost of introducing these technologies into practice (20.5 %) (Fig. 4).

Finally, when asked about additional support needed, 56.1 % of doctors requested more specific training courses, while 22 % expressed a need for technical support (Fig. 4C).

## 4. Discussion

A survey assessing medical attitudes toward AI use in FM was conducted during the ATLAS 2024. The main results revealed that before the congress, a considerable portion of participants (46.2 %) had read scientific articles about the use of AI in clinical practice, demonstrating an existing interest in AI. Interestingly, in a web-based cross-sectional study, 84.1 % of participants were aware of AI; among them, 61.1 % believed AI to be a tool that aids healthcare professionals, while 12.5 % expressed concerns that AI might replace physicians, pharmacists, or nurses in the healthcare system [13].

In our investigation, we found that practical implementation of AI tools was limited, with only 15.4 % of respondents having used them in practice. This gap between theoretical knowledge and practical application underscores the challenges in adopting new technologies in clinical settings. Notably, AI technologies often provide integration with existing clinical workflows, electronic health records (EHRs), and patient management systems. This integration can be complex and time-consuming, requiring technical expertise and support.

When asked about the main barriers to acquiring sufficient knowledge in the field, the majority cited a lack of adequate training (42.9 %). This aligns with the most reported concern (27.3 %) and the most requested support by professionals, namely the opportunity to attend specific training courses (52.3 %). These findings highlight the need for practical courses to help doctors implement AI technologies in clinical practice, as 61.5 % reported feeling incapable of doing so in their daily practice. Additionally, practical courses could address the resistance to change, which was identified as the second most perceived barrier (24.5 %). Probably, in different clinical contexts, by introducing more hands-on training, clinicians would be better equipped to apply AI for early diagnosis, decision support, and personalized treatment.

Professionals also expressed concern about interpreting AI-generated results and how to communicate these to patients (27.3 %). The ability to explain data results derived from AI systems is a significant topic of debate, particularly with artificial neural networks and their black-box nature [14]. It is crucial to have more explicable systems to ensure their results can be clinically validated, as professionals will have a better understanding of how these systems function. Furthermore, AI explainability promotes shared decision-making in patient-clinician discussions, enhancing patients’ understanding and perception of risks, thereby helping them feel more informed and confident in their treatment choices. Our study results are consistent with findings from a recent scoping review on factors influencing the acceptability of AI in medical imaging domains among healthcare professionals. The review identified various categories, including user factors related to trust, understanding of AI systems, receptiveness to technology, and AI literacy [15].

Regarding AI literacy, the ATLAS AI Lab had a positive impact on participants’ perceptions of AI in FM management. The majority (57.7 %) reported an improved perception of AI, with a smaller group (7.7 %) noting a much-improved view. However, 26.9 % reported no change, and one doctor (3.8 %) had a worsening view. This mixed response highlights the need for ongoing education and support to address concerns and skepticism [16]. Despite improved perceptions, a significant number of participants (61.5 %) felt poorly prepared to integrate AI tools into clinical practice. This indicates a substantial gap between recognizing the potential of AI and feeling competent to use it effectively. Understanding of AI’s usefulness in clinical practice improved in 38.5 % of respondents, while 50.5 % reported a partial improvement. However, the ability to distinguish between different types of AI algorithms remained low, with 42.3 % unable to distinguish and 50 % only partially able to do so. This suggests that while awareness is increasing, deeper technical understanding is still lacking.

While the results are highly intriguing, they should be interpreted with caution. The major limitation is the small sample size. However, the use of a well-defined and representative sample, along with a controlled administration context, partially mitigates this limitation [11,12]. Additionally, although the sample size is small, our study was exploratory, aiming to capture initial insights into clinicians’ perceptions of AI in FM management. Specifically, due to the type of sampling, the sample size was not determined through a pre-calculated method. Therefore, no formal sample size calculation was performed, and the number of participants was based on the availability of the attendees at the conference. As a result, the findings reflect the opinions and experiences of this specific group of participants and do not necessarily represent the entire population of physicians. Therefore, our goal was not to generate definitive conclusions but rather to gather preliminary data on medical professionals’ perceptions of AI in fibromyalgia management. This initial investigation aims to identify trends and areas of concern, which can inform larger, more statistically powered investigations.

## 5. Conclusion

Despite a generally positive shift in perception following the congress, many doctors still feel unprepared and lack the necessary knowledge to effectively utilize AI tools. Addressing barriers such as insufficient training, resistance to change, and cost concerns is crucial. Therefore, the survey results underscore the need to introduce more specific and practical courses to increase the clinicians’ knowledge of AI. It is a key step to integrate AI technologies in healthcare and to effectively assist patients with different clinical conditions including FM. Additionally, these AI systems need to be interpretable and explainable by both professionals and patients to enhance the doctor-patient relationship.

## Figures and Tables

**Table 1:** Questionnaire.

**Demographic information**
1. Age < 30 30–39 40–49 50–59 60+
2. Sex M F Other I prefer not to respond
3. Profession Physiatrist Rheumatologist Neurologist Family Medicine Doctor Other (please specify)
4. Type of work Autonomous Dependent (public) Dependent (private)
5. Years of clinical experience < 5 years 5–10 years 11–20 years > 20 years
6. Geographic area Northern Italy Center Italy Southern Italy and Islands
**Perception of artificial intelligence in the management of fibromyalgia**
7. Before the congress, had you already read scientific articles related to the use of AI in clinical practice? Yes No
8. Before the congress, had you already used AI tools in your clinical practice? Yes No
9. If so, what tools did you use? Diagnostic imaging Decision support systems Patient monitoring Chatbots Other (please specify)
10. What do you believe are the main barriers to the adoption of AI in clinical practice? (Select all applicable options) Lack of knowledge/training Resistance to change High costs Data security issues Uncertainty about reliability
11. Before participating in this conference, what was your level of knowledge about the use of artificial intelligence (AI) in the management of fibromyalgia? No knowledge at all Limited Moderate Advanced Experienced
12. How much has your perception of the usefulness of AI in the management of fibromyalgia changed since attending the congress? Much worsened Worsened No change Much improved
13. What aspects of AI do you find most promising for improving the management of fibromyalgia? (Select all applicable options) Early diagnosis Identification of biomarkers and other signatures Personalization of treatment Symptom monitoring Prediction of symptom fluctuations Decision support for doctors
14. What are your main concerns about the use of AI in the management of fibromyalgia? (Select all applicable options) Data accuracy Data security Patient understanding and interpretation of results Insufficient training for physicians Cost of AI technologies Other (please specify)
15. After the congress, how prepared do you feel to integrate AI-based tools into your clinical practice for the management of fibromyalgia? Not at all prepared Slightly prepared Moderately prepared Very prepared Fully prepared
16. Have you gained a clearer understanding of what “machine learning” is and how it can be in the management of fibromyalgia since the congress? Yes No Partly
17. Would you be able to distinguish between different types of algorithms used in artificial intelligence, such as supervised learning, unsupervised learning, and reinforcement learning? Yes No Partly
18. Do you have a clear understanding of what a “neural network” is after the congress? Yes No Partly
19. Would you be able to explain the importance of neural networks in data analysis and outcome prediction in the management of fibromyalgia? Yes No Partly
20. Did you have the opportunity to discuss with colleagues or experts during the congress to clarify doubts or deepen your understanding of these concepts? Yes No Partly
21. What additional information or support would you find useful in integrating AI into the management of fibromyalgia? (Select all applicable options) Specific training courses Clinical guidelines Technical support Clinical case studies Other (please specify)

## Data Availability

The data that support the findings of this study are available from the corresponding author upon reasonable request.
